# The Role of Fluid Mechanics in Coronary Atherosclerotic Plaques: An Up-to-Date Review

**DOI:** 10.31083/j.rcm2502049

**Published:** 2024-01-29

**Authors:** Yaoming Yang, Yang Song, Xiaolin Mu

**Affiliations:** ^1^Department of Radiology, Central Hospital of Dalian University of Technology, 116033 Dalian, Liaoning, China; ^2^Department of Graduate School, Dalian Medical University, 116000 Dalian, Liaoning, China

**Keywords:** coronary artery, fluid mechanics, plaques, wall shear stress, axial plaque stress, fractional flow reserve

## Abstract

Most acute coronary syndromes are due to a sudden luminal embolism caused by the rupturing or erosion of atherosclerotic plaques. Prevention and treatment of plaque development have become an effective strategy to reduce mortality and morbidity from coronary heart disease. It is now generally accepted that plaques with thin-cap fibroatheroma (TCFA) are precursors to rupturing and that larger plaques and high-risk plaque features (including low-attenuation plaque, positive remodeling, napkin-ring sign, and spotty calcification) constitute unstable plaque morphologies. However, plaque vulnerability or rupturing is a complex evolutionary process caused by a combination of multiple factors. Using a combination of medicine, engineering mechanics, and computer software, researchers have turned their attention to computational fluid mechanics. The importance of fluid mechanics in pathological states for promoting plaque progression, inducing plaque tendency to vulnerability, or even rupture, as well as the high value of functional evaluation of myocardial ischemia has become a new area of research. This article reviews recent research advances in coronary plaque fluid mechanics, aiming to describe the concept, research implications, current status of clinical studies, and limitations of fluid mechanic’s characteristic parameters: wall shear stress (WSS), axial plaque shear (APS), and fractional flow reserve (FFR). Previously, most computational fluid dynamics were obtained using invasive methods, such as intravascular ultrasound (IVUS) or optical coherence tomography (OCT). In recent years, the image quality and spatial resolution of coronary computed tomography angiography (CCTA) have greatly improved, making it possible to compute fluid dynamics by noninvasive methods. In the future, the combination of CCTA-based anatomical stenosis, plaque high-risk features, and fluid mechanics can further improve the prediction of plaque development, vulnerability, and risk of rupturing, as well as enabling noninvasive means to assess the degree of myocardial ischemia, thereby providing an important aid to guide clinical decision-making and optimize treatment.

## 1. Introduction

Coronary atherosclerotic heart disease is one of the most common cardiovascular diseases and is the number one cause of death worldwide. Most acute coronary syndromes are the result of sudden intraluminal embolism caused by either the rupturing or erosion of atherosclerotic plaques, and there may be no signs or warnings before an acute attack. The only way to effectively reduce the burden of cardiovascular disease and reduce mortality and morbidity is to prevent acute coronary events (including acute myocardial infarction and sudden cardiac death). However, the use of cardiovascular imaging to determine whether a patient is on the verge of an acute coronary event is a challenge and needs to be addressed.

**Fig. 1.**
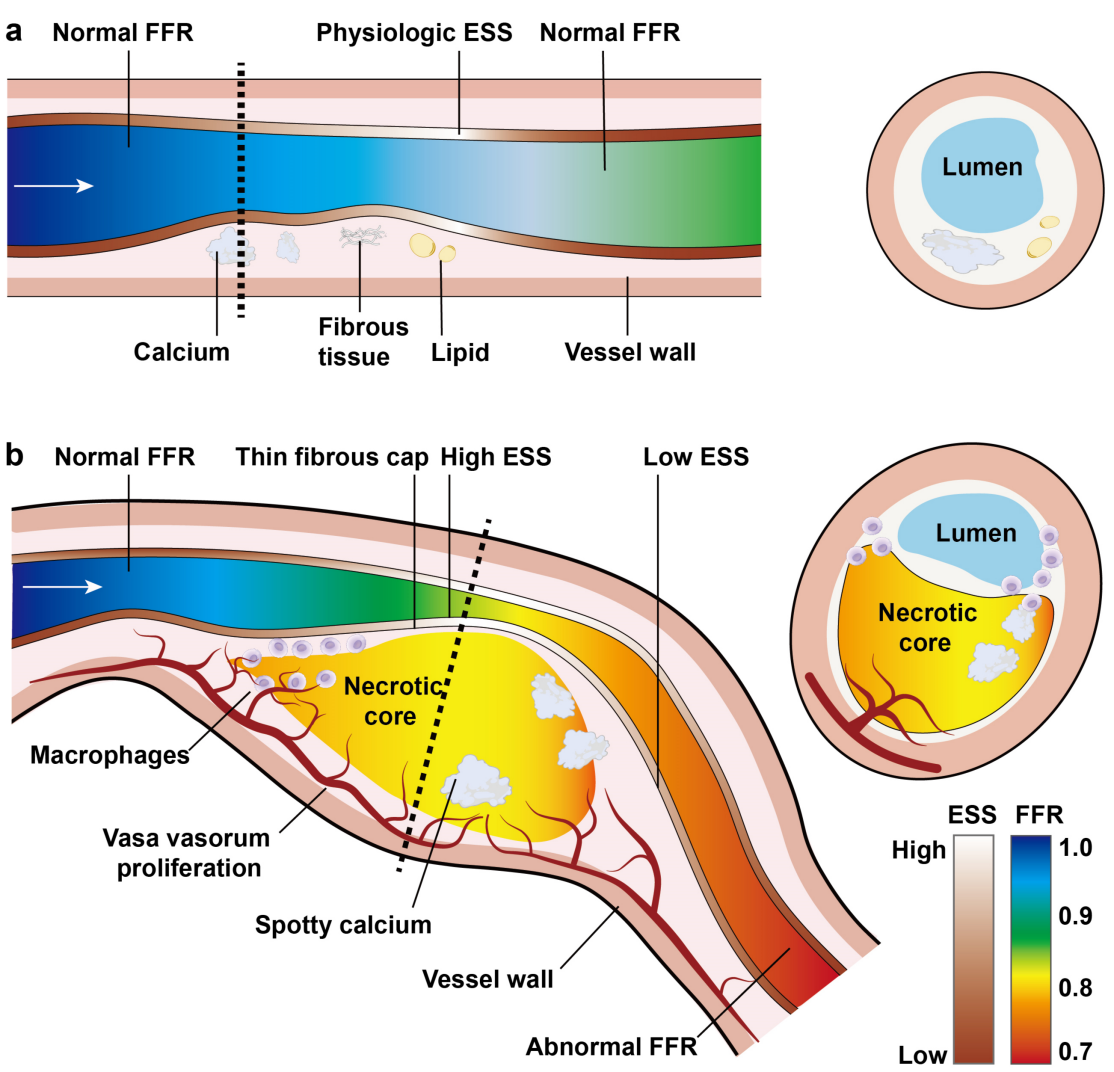
**Morphological and fluid mechanics characteristics of stable (a) and vulnerable (b) plaques**. ESS, endothelial shear stress, also known as wall shear stress (WSS); FFR, fractional flow reserve.

In recent years, researchers have extensively explored the development of coronary atherosclerotic plaque characteristics, early intervention to slow plaque progression, and methods to promote plaque regression, and effectively reduce the occurrence of major adverse cardiovascular events [1, 2, 3]. Rupture-prone plaques have a morphology and fluid mechanics distinct from stable plaques (See Fig. 1). Intravascular ultrasound (IVUS) or optical coherence tomography (OCT)-based studies have shown that the characteristics of plaques covered with a thin-cap fibroatheroma (TCFA), and certain fluid mechanical characteristics are associated with the development of major adverse cardiovascular events (MACE) [4], However, these studies are invasive, expensive, and not always indicated, making them difficult to be widely performed as a screening tool in clinical practice.

Using the combination of medical and engineering mechanics and computer post-processing software, more and more studies are focusing on computational fluid dynamics to investigate the potential impact of biological forces on atherosclerotic plaques and to assess the blood supply from a functional perspective [5]. The greatly improved image quality and spatial resolution of coronary computed tomography angiography (CCTA) have made a noninvasive approach to computational fluid dynamics possible. In the future, the combined use of multiple imaging techniques will be more beneficial for the early diagnosis of acute coronary events. In this review, we provide an overview of computational fluid dynamics characteristics, including wall shear stress (WSS), axial plaque stress (APS), fractional flow reserve (FFR), and the relationship between coronary atherosclerotic plaque formation and progression, vulnerability, and rupturing, and the current status and limitations of clinical studies on functional assessment of myocardial ischemia.

## 2. Coronary artery WSS

### 2.1 Concept

WSS, also known as endothelial shear stress (ESS), is the tangential force generated by viscous blood on the vascular endothelium, i.e., the parallel frictional force exerted by blood flow on the endothelial surface, which can participate in and contribute to the local inflammatory response, as well as to the pathophysiological processes that promote the development, progression, or stabilization of coronary atherosclerosis. Normal values of WSS in the physiological state are in the range of 1–2.5 Pa [6, 7]. The magnitude of WSS can be interchanged using various units, e.g., 1 Pa = 1 ${\mathrm{N/m}}^{2}$ = 10 ${\mathrm{dynes/cm}}^{2}$.

### 2.2 Clinical Significance

WSS is a hemodynamic factor whose magnitude and direction are related to many factors, such as blood velocity, blood viscosity, interbranch flow, the state of the distal vessels (including the microcirculation), and the geometry of the lumen, alongside continuous changes in the cardiac cycle [8]. Vascular endothelial cells have real-time detection of WSS pressure receptors, which in turn activate complex endothelial regulatory pathways [9]. In the flat part of the coronary tree, where the lumen geometry is uniform and the flow direction is homogeneous, the WSS is often within the physiological range and stimulates the endothelial cells to continuously release nitric oxide (NO), an important component in the regulation of vascular tone and blood flow distribution, which has strong anti-apoptotic, anti-inflammatory, anti-platelet aggregation, and promotes vascular growth and regeneration. Moreover, it is known as an endogenous platelet aggregator and adhesion inhibitor, thereby avoiding the development of atherosclerosis. In contrast, the lumen geometry is heterogeneous, and the direction of blood flow varies at the bifurcations and bends of the coronary tree and in the post-functional stenosis region, the size and direction of the WSS are altered, thereby making the coronary arteries in this segment susceptible to endothelial damage and reduced NO production, leading to reduced anti-inflammatory and anti-platelet aggregation capacities, and the promotion of the early development of atherosclerotic plaques [10].

### 2.3 Current Status of Clinical Studies

Although the risk factors for plaque formation (including smoking, high cholesterol, hypertension, and insulin resistance) are theoretically thought to affect the entire vascular bed, there are specific sites in the coronary arteries (e.g., outer walls of bifurcated vessels, lateral branches, and inward bends) that interfere with normal flow and lead to plaque formation [11]. Feng *et al*. [12] combined two computational fluid dynamics (CFD) models with computed tomography imaging and found that three key regions around the bifurcation, including the bifurcation ridge and the medial and lateral walls of the bifurcation, are prone to atherosclerosis formation.

#### 2.3.1 Relationship between Low WSS and High-Risk Plaques

Numerous studies have shown that coronary artery walls with low WSS (<1 Pa) segments are more prone to atherosclerosis and promote the development of high-risk plaques. Hoogendoorn *et al*. [13] evaluated coronary artery high-risk plaque characteristics by IVUS in animal experiments and found that low WSS was an independent predictor of high-risk plaque development and that the severity of the high-risk plaque characteristics was significantly correlated with the degree of low WSS, which lead to the conclusion that the magnitude of low WSS determines the atherosclerotic lesion complexity and heterogeneity, and predicted high-risk plaque development. In a European cardiology study, WSS measured by OCT found that luminal dilated remodeling and localized low WSS were strongly associated with high-risk plaques and that the frequency of vulnerable plaques, and the probability of acute coronary syndrome (ACS) was increased in segments of coronary arteries with increased WSS scores [14]. Corban *et al*. [15] performed a 6-month follow-up of 20 patients with non-obstructive coronary artery disease, measuring WSS by IVUS, and found that the combination of plaque load, WSS, and plaque phenotype had incremental values in predicting coronary plaque progression and plaque vulnerability. In addition, low WSS segments were prone to larger plaques, the progression of necrotic cores, and negative remodeling compared with normal WSS segments [7]. In conclusion, low WSS is currently considered an independent predictor of high-risk plaque development and correlates with the severity of high-risk plaques.

#### 2.3.2 Relationship between High WSS and High-Risk Plaques

Studies have shown that high WSS (>2.5 Pa) promotes the destabilization of high-risk plaques, making them more prone to rupturing. Previous studies have suggested that high WSS segments in the coronary wall may prevent the development of atherosclerosis [6, 16]. However, recent studies have found that the location of the plaque rupture with prolonged atherosclerosis tends to correlate with high WSS [17]. Plaques in long-term high WSS segments tend to transform into a more fragile, higher-risk phenotype, i.e., a larger core of stromal lipid necrosis, intraplaque hemorrhage [18], increased calcium load, degeneration of fibrous and fibrofatty tissue, positive remodeling [7], and napkin-ring sign [19]. Park *et al*. [20] obtained WSS from CCTA data of 80 patients by CFD reconstruction and showed that the proportion of high-risk plaques exposed to high WSS segments was significantly increased, while also demonstrating that the CCTA-based CFD approach allows for the noninvasive measurement of the coronary plaques affecting the WSS. A study by Okamoto *et al*. [21] further confirmed that high WSS is a predictor of independent risk factors for thin-fibrous cap atherosclerotic plaques under OCT. In addition, another study, which included 411 patients with stable coronary artery disease and hemodynamic abnormalities, found that higher WSS in proximal coronary plaque segments was a predictive risk factor for myocardial infarction [22].

### 2.4 Limitations

There are difficulties in measuring WSS, which is mainly obtained by invasive IVUS or OCT methods, such as it is invasive, expensive, complicated to operate, difficult to promote and popularize; moreover, it is impossible to measure WSS directly in the physiological state, and there is a lack of a unified modeling method for WSS based on CCTA calculations.

## 3. Coronary Artery APS

### 3.1 Concept

APS is the axial component of the stress acting on the plaque, which is an independent pressure equal to the combined force of all types of stresses acting on the central line of the coronary artery. The pressure value of APS is much higher than ESS. In the area of plaque stenosis and the state of myocardial hyperemia, ESS reaches its maximum value, yet APS still exceeds it by more than 40 times [23]. This is mainly related to the absolute pressure on the plaque surface.

### 3.2 Clinical Significance

It was found that APS can both directly participate in plaque rupturing, especially downstream of atherosclerotic plaques [24] and can reflect plaque geometry [23]. It also serves as a link between hemodynamics and function.

### 3.3 Current Status of Clinical Studies

Presently, few studies have been performed on APS. Toba *et al*. [25] divided a total of 47 lesions in 20 patients into three groups: normal vessel walls (group N), thick-walled fibrous plaques without membranes (group F), and plaques with lipid or plaque calcification (group L). By calculating WSS and APS, the results showed that group N had the highest WSS, while APS was significantly lower than the other two groups. Multifactorial analysis adjusting for stenosis severity showed that low APS was independently associated with group N, while high APS was independently associated with group L, thereby leading to the conclusion that APS may influence the onset and progression of coronary atherosclerosis and improve the prediction of lesion characteristics. Choi *et al*. [23] analyzed 114 lesion vessels (81 patients) based on CCTA images and calculated plaque axial stress (APS) by extracting the axial component of the fluid mechanics stress acting on the stenotic lesion, classifying the lesions into upstream dominant lesions (upstream radius gradient (RG) > downstream RG) and downstream dominant lesions (downstream RG < upstream RG) by RG. The APS was found to be an independent feature of the stenotic segment and strongly correlated with lesion geometry: upstream APS increased linearly with lesion severity, whereas downstream APS exhibited a concave function with lesion severity, i.e., it appeared to decrease before increasing. In addition, APS was negatively correlated with lesion length, which may explain the higher risk of rupturing in short or focal plaques than in diffuse plaques. These hemodynamic and geometric indices may help in the clinical assessment of the risk of future plaque rupturing and in determining treatment strategies for patients with coronary artery disease.

### 3.4 Limitations

Current research and pathophysiological understanding of WSS far exceeds that of APS; however, the role that APS may play in plaque ruptures is much more important than that of WSS. As mentioned previously, the measurement of APS remains limited owing to the complex and invasive nature of the technique.

## 4. Coronary FFR

### 4.1 Concept

Under normal physiological conditions, there is no obvious resistance when blood flows through the epicardial coronary artery, with the main resistance coming from the microcirculation. In clinical practice, vasodilators induce maximum myocardial microcirculation congestion, in this condition, myocardial blood flow is only affected by perfusion pressure, and the change in perfusion pressure caused by stenosis can reflect the change in blood flow. FFR is the ratio of the maximum blood flow obtained in the region of the myocardium supplied by this vessel in the presence of epicardial coronary stenosis compared to the maximum blood flow obtained in the same region under normal conditions This is defined as the ratio of the average pressure distal to the average pressure proximal to the stenosis in a state of myocardial hyperemia. It is obtained from a pressure transducer during coronary angiography and is considered the gold standard for the evaluation of functional ischemia in coronary artery disease [26]. FFR-guided percutaneous coronary intervention (PCI) significantly improves the prognosis of stable coronary artery disease [27].

Computed tomography-fractional flow reserve (CT-FFR) is a computational model of fluid dynamics, which is applied to routinely standardized CCTA images to simulate and calculate the hemodynamic differences at the stenosis in the physiological state of the coronary artery and to provide a simulated invasive FFR value.

### 4.2 Clinical Significance

A series of studies [28, 29, 30, 31, 32, 33] have confirmed that FFR values based on CCTA simulations are in good agreement with invasive FFR values, providing a reliable reference for the presence of myocardial ischemia and the need for hemodynamic reconstruction in patients with coronary artery disease. In addition, in a recent multicenter study [34], the sensitivity, specificity, and accuracy of the new uCT-FFR software in identifying myocardial ischemia were found to be 0.89, 0.91, and 0.91, respectively, based on the invasive FFR values being the gold standard [35, 36].

### 4.3 Current Status of Clinical Studies

Currently, an invasive FFR ≤0.70 is considered specific for myocardial ischemia and is recommended for revascularization, while an FFR >0.80 is rarely associated with myocardial ischemia and is recommended for conservative pharmacological treatment [37]. Patients with FFR values between 0.70 and 0.80 are considered to be in a “gray zone” and additional factors need to be considered to determine whether revascularization is appropriate, or if they should be treated with medications only. Further, it should be considered whether their combined death, the risk of combined death, or myocardial infarction and total death is twice as high as those only treated with medication alone [38, 39]. The current thresholds for invasive FFR also apply to CT-FFR and have generated extensive discussion for patients with CT-FFR values between 0.7 and 0.8. The 2022 Chinese Society of Radiology expert consensus document [40] indicates that more factors need to be considered, including symptoms (especially severity of chest pain), risk factors, plaque location, whether it is a high-risk plaque, ΔCT-FFR, area of myocardial blood supply, and functional test results, such as myocardial perfusion imaging. In addition, the gender of the patient should also be considered. A 2018 study showed that CT-FFR differed between genders, with women having higher CT-FFR values at the same degree of stenosis [41].

CT-FFR is not only a good guide for clinical decision-making but also for the evaluation of plaque progression and the prediction of future major adverse cardiovascular events. Yu *et al*. [35], in a prospective study following patients treated with statins, found that in non-calcified plaques, the Δ CT-FFR values decreased as the plaque volumes decreased. There were significant differences in the number of high-risk plaques in different FFR ranges, with the number of high-risk plaques increasing as the FFR decreased, while both FFR values and high-risk plaque characteristics were significantly associated with poor prognosis over five years [36]. Furthermore, it has been shown that CT-FFR has better efficacy in predicting coronary events compared to clinical risk factors [42]. In a large prospective international multicenter study, 1592 subjects with negative CT-FFR values did not experience death, myocardial infarction, or unplanned hospitalization for acute coronary syndrome and emergency revascularization within 90 days [43]. The ADVANCE (Assessing Diagnostic Value of Non-invasive FFRCT in Coronary Care) investigation [44] found that at the one-year follow-up, all CT-FFR-negative patients had a reduced proportion of revascularizations, fewer MACE events, and significantly fewer cardiac deaths or myocardial infarctions compared to patients with positive CT-FFR values. At short or long-term follow-ups, patients with positive CT-FFR values were more likely to have MACE compared with CT-FFR-negative patients [43, 44]. Yang *et al*. [45] showed that patients with CT-FFR ≤0.70 had a 2.4-fold increase in the development of MACE.

The ΔCT-FFR has been defined as the difference between proximal and distal CT-FFR values. Additionally, it has been suggested that a ΔCT-FFR ≥0.06 may be a better predictor of unstable lesions in ACS compared with the CT-FFR measurement of distal lesions, thereby directly reflecting the decrease in CT-FFR values along the focal zone vessels [46]. This study also showed that for the identification of vulnerable vessels with tandem plaques, the ΔCT-FFR had the highest C-index (concordance index) among the four combinations of hemodynamic variables CT-FFR, ΔCT-FFR, WSS, and APS, i.e., it showed stronger predictive efficacy [46]. Therefore, the introduction of the ΔCT-FFR can more accurately assess the lesion-specific hemodynamic significance in the presence of tandem lesions, and, this parameter can also provide some predictive value for poor prognoses.

The current application of CT-FFR for the guidance of PCI or coronary artery bypass grafting (CABG) can be used as an experimental tool to determine its relevance for additional diseases that affect coronary fluid mechanics.

### 4.4 Limitations

CT-FFR has many shortcomings in clinical applications. The best indication is for patients with CCTA presenting a luminal stenosis of 30%–90% without complex lesions, while its application in myocardial bridges, complex coronary artery disease, severe aortic stenosis, prosthetic bioprosthesis implantation, and revascularization history (PCI, CABG) is limited, while its accuracy is affected by the extensive calcification of the coronary artery wall. A meta-analysis showed that the specificity of CT-FFR decreased with the increase of coronary artery calcium (CAC). Here, CAC = 400 and CAC = 1000 were two very important cutoff values, whereby both indicated an increase in the CT-FFR false-positive rate [47]. In patients with extensive coronary calcification, loading CT myocardial perfusion may be more appropriate than CT-FFR [48].

## 5. Outlook

Although CCTA can detect morphological features of high-risk plaques, it is limited by the spatial distribution rate. Thus, its inability to detect fibrous cap thickness or histological features of plaque rupture, which may be better visualized by coronary MR imaging, means that the use of CCTA needs to be further evaluated by large prospective trials. In addition, exploring novel contrast agents to obtain plaque metabolic information could also improve the assessment of plaque vulnerability by CCTA.

However, a hydrodynamic model based on CCTA simulations has not yet been established. Moreover, large-scale medical-industrial studies combined with longitudinal imaging tests are still needed to obtain standardized fluid mechanics as reference indicators.

To obtain multiparameter information on plaques quickly and efficiently, an AI-based automated plaque assessment tool is essential and needs to be further developed.

The mechanism of plaque onset, progression, and rupturing is complex and influenced by several factors. The detailed process of hydrodynamic influence on plaque progression or regression has not yet been continuously observed. Additional prospective trials are needed to obtain information on this technique. The noninvasive assessment method of CT-FFR functional science has the potential to broaden the application of CCTA. However, further studies are needed to confirm the application of this methodology for complex cardiovascular diseases.

## 6. Conclusions

Fluid mechanics play an extremely important role in coronary atherosclerotic plaques. The combination of individual plaque morphology and functional parameters can provide new ways of detecting vulnerable and fragile plaques, as well as evaluating functional myocardial ischemia in coronary artery disease, thereby facilitating the early diagnosis of potential acute coronary events.
